# Gestational trophoblastic disease: does central nervous system chemoprophylaxis have a role?

**DOI:** 10.1038/sj.bjc.6690203

**Published:** 1999-03

**Authors:** A M Gillespie, N Siddiqui, R E Coleman, B W Hancock

**Affiliations:** Gestational Trophoblastic Disease Screening and Treatment Centre, Yorkshire Cancer Research, Weston Park Hospital, Whitham Road, Sheffield, S10 2SJ, UK; Department of Obstetrics & Gynaecology, Southern General Hospital, 1345 Govan Road, Glasgow, G51 4TF, UK

## Abstract

In the UK there are standardized surveillance procedures for gestational trophoblastic disease. However, there are differences in practice between the two treatment centres in terms of definition of persistent gestational trophoblastic disease, prognostic risk assessment and chemotherapeutic regimens. The role of prophylactic chemotherapy for cerebral micrometastatic disease in persistent gestational trophoblastic disease is unclear. We have analysed the outcome of 69 patients with lung metastases who elsewhere might have received prophylactic intrathecal chemotherapy. Of the 69 patients, 67 received intravenous chemotherapy only. The other two patients had cerebral metastases at presentation. One patient who received only intravenous chemotherapy subsequently developed a cerebral metastasis, but this patient's initial treatment was compromised by non-compliance. This experience supports our current policy of not treating patients with pulmonary metastases, without clinical evidence of central nervous system (CNS) involvement, with prophylactic intrathecal therapy. © 1999 Cancer Research Campaign


*British Journal of Cancer* (1999) **79**(7/8), 1270–1272
© 1999 Cancer Research Campaign
Article no. bjoc.1998.0203

### Gestational trophoblastic disease: does central nervous system chemoprophylaxis have a role?

**AM Gillespie[1], N Siddiqui[2], RE Coleman[1] and BW Hancock[1]**

[1]Gestational Trophoblastic Disease Screening and Treatment Centre, Yorkshire Cancer Research, Department of Clinical Oncology, Weston Park Hospital, Whitham Road, Sheffield S10 2SJ; [2]Department of Obstetrics & Gynaecology, Southern General Hospital, 1345 Govan Road, Glasgow G51 4TF

**Summary** In the UK there are standardized surveillance procedures for gestational trophoblastic disease. However, there are differences in practice between the two treatment centres in terms of definition of persistent gestational trophoblastic disease, prognostic risk assessment and chemotherapeutic regimens. The role of prophylactic chemotherapy for cerebral micrometastatic disease in persistent gestational trophoblastic disease is unclear. We have analysed the outcome of 69 patients with lung metastases who elsewhere might have received prophylactic intrathecal chemotherapy. Of the 69 patients, 67 received intravenous chemotherapy only. The other two patients had cerebral metastases at presentation. One patient who received only intravenous chemotherapy subsequently developed a cerebral metastasis, but this patient's initial treatment was compromised by non-compliance. This experience supports our current policy of not treating patients with pulmonary metastases, without clinical evidence of central nervous system (CNS) involvement, with prophylactic intrathecal therapy.

The establishment of a UK Trophoblastic Disease Registration and Surveillance Scheme by The Royal College of Obstetricians & Gynaecologists and The Department of Health in 1973 has been a great success. Following diagnosis, all patients with gestational trophoblastic disease (GTD) are registered with and followed up by one of three centres located at: Weston Park Hospital, Sheffield; Charing Cross Hospital, London; and Ninewells Hospital, Dundee. Follow-up takes the form of serial urinary and serum human chorionic gonadotrophin (hCG) measurements. Standardized surveillance procedures are adopted nationwide. Patient compliance with the follow-up programme is very high, which allows the two treatment centres (in Sheffield and London) to adopt stringent treatment criteria and a conservative treatment philosophy. The UK treatment centres report high cure rates while exposing the minimal number of patients possible to cytotoxic chemotherapy (Sheridan et al, 1993).

As standardized surveillance procedures are adopted nationwide, there are several differences in practice between the two treatment centres in terms of definition of persistent GTD, initial prognostic risk assessment and chemotherapeutic regimens. One difference is the use of prophylactic chemotherapy for possible occult central nervous system (CNS) metastases. It is well recognized that choriocarcinoma can metastasize to the brain. Charing Cross Hospital has previously reported that patients with persistent GTD and multiple pulmonary metastases or other high-risk features are at increased risk of CNS metastases. Since 1973 their patients without evidence of CNS metastases, but with pulmonary metastases, have been given CNS chemoprophylaxis in the form of intrathecal (i.t.) methotrexate (12.5 mg) with their first three cycles of systemic chemotherapy (Althanassiou et al, 1983; Newlands, 1997). At Weston Park Hospital, however, our management differs in that only patients with proven CNS involvement are treated with CNS chemotherapy. Our policy is to perform a computerized tomographic (CT) scan of the head and a lumbar puncture (providing there is no clinical evidence of raised intracranial pressure) to obtain cerebrospinal fluid (CSF) for βhCG levels in all patients who: (1) are classified 'high risk' at initial prognostic risk assessment by the Sheffield modification of the Charing Cross Hospital prognostic scoring system (≥ 8 points – Sheridan et al, 1993); (2) have βhCG levels of 50 000 iu $l^{-1}$ or greater, and/or (3) have five or more metastases visible on chest radiographs/eight or more nodules visible on CT scan of thorax. Confirmation of CNS involvement is established by imaging and/or a high (> 1:60) CSF–serum hCG ratio (Bagshawe and Harland, 1976).

The cure rates for patients with GTD are now high, and so the focus of patient care has shifted from defining active chemotherapeutic agents to optimizing therapeutic regimens and minimizing risk to patients diagnosed with this condition. Our objective in this study was to evaluate the results of our conservative treatment approach to putative occult CNS disease in patients with GTD and pulmonary metastases.

### METHODS

### Subjects

All patients with GTD registered between 1987 and 1996 were included in this retrospective analysis. Of 195 patients with persistent GTD who were treated in this time period, 69 had pulmonary metastases at initial presentation detected by chest radiography and/or CT of the thorax. The median age of patients in this group was 27 years (range 16–49 years).

### Treatment

Treatment regimens employed at our centre are reported elsewhere (Fisher and Hancock, 1997). Treatment of patients with established CNS metastases involves a combination of systemic chemotherapy

*Received 24 February 1998*
*Revised 30 June 1998*
*Accepted 27 July 1998*

*Correspondence to:* AM Gillespie and i.t. methotrexate. The systemic therapy comprises a two-arm regimen, with 7-day intervals between each arm, which is continued for 2 months after normalization of serum hCG levels. Arm A consists of methotrexate 1 g $m^{-2}$ over 24 h with folinic acid rescue. Arm B consists of dactinomycin 0.5 mg and etoposide 100 mg $m^{-2}$ 6 h apart, daily for 3 days each cycle. Methotrexate (12.5 mg) i.t. is administered once during arm B of each treatment cycle and is continued for four injections after the CSF–serum ratios have returned to normal. A combination of high-dose intravenous methotrexate and i.t. methotrexate is used to ensure adequate CNS penetration of this drug (Shapiro et al, 1975).

### RESULTS

### Pulmonary metastases

Sixty-nine patients were identified with pulmonary metastases. Chest radiography and CT of the thorax were performed in 63 cases and CT only in the remaining six cases. Among the 63 patients in whom both investigations were performed, 36 had metastases detected by both modalities and 27 had metastases detectable on CT only.

### Prognostic risk scores, treatment and follow-up

Patients were assessed according to modified Charing Cross criteria (Sheridan et al, 1993). The median risk score was 6 (range 1–23). Fifty-one patients were classified as having low-risk disease and 18 as having high-risk disease. Thirty-one low-risk patients were successfully treated with first-line therapy (low-dose methotrexate); 19 required second-line chemotherapy with dactinomycin and etoposide. Treatment is ongoing in one patient. Of the 18 high-risk patients, 11 were cured with first-line therapy (methotrexate/dactinomycin/etoposide), three with second-line therapy (cisplatin/etoposide/cyclophosphamide) and one with the CNS regimen (see above); one further patient was inappropriately but successfully treated with the low-risk regimen. Two patients in the high-risk prognostic group have died from disease. Sixty-six patients have successfully completed their treatment with a median follow up of 62 months (range 14–128 months).

### CNS metastases

Three patients with persistent GTD received treatment for CNS metastasis. The first patient had an initial risk score of 7 and was classified as having low-risk disease. Chest radiography was negative but CT scanning showed multiple small pulmonary metastases. This patient's prognosis was compromised by late presentation and non-compliance with follow-up and she subsequently developed a solitary cerebral metastasis, which was treated with neurosurgery, CNS chemotherapy and on further relapse with high-dose chemotherapy. Treatment is ongoing for active, probably drug-resistant, pulmonary disease. The second patient had disseminated postpartum choriocarcinoma with CNS involvement at presentation and was successfully treated with nine cycles of the regimen described above. She is alive and disease free at 36 months. The final patient had a placental site trophoblastic tumour with CNS involvement and died of disease despite multiple therapeutic interventions.

### DISCUSSION

There are differing policies world-wide for the treatment of potential and established CNS disease in GTD. In the UK, surgical and chemotherapeutic treatment modalities are generally used in patients with established CNS metastases (Althanassiou et al, 1983; Rustin et al, 1989). In contrast, GTD has been shown to be radiosensitive (Brace, 1968), and elsewhere the policy of whole-brain irradiation in conjunction with chemotherapy has met with some success (Weed and Hammond, 1980; Yordan et al, 1987; Evans et al, 1995). However, CNS metastasis still causes significant mortality, and those in this patient group have a poorer prognosis than the rest of the GTD patient population.

In view of the difficulties inherent in the treatment of established CNS disease it would seem logical to attempt to identify patients at risk of CNS metastases and try to prevent this problem arising. This is the basis of the policy of CNS prophylaxis used at Charing Cross Hospital. However, the administration of i.t. chemotherapy is not without risk and should only be recommended when it is absolutely necessary and of proven value. This study suggests that prophylactic i.t. methotrexate is unnecessary in patients with pulmonary metastases. This study is supported by published data from North America which demonstrate that the cure rate for patients with pulmonary metastases not receiving CNS chemoprophylaxis approaches 100% (Du Beshter et al, 1991; Soper et al, 1994; Roberts and Lurain, 1996).

Also, it is clear from this retrospective analysis that, where a successful surveillance scheme is in operation, patients with persistent GTD necessitating chemotherapy are generally identified before widespread pulmonary metastasis occurs. Of the 195 patients treated during the study time period, only 69 (35%) had pulmonary metastases.

In conclusion, restriction of IT treatment to those patients with established CNS disease appears to be safe and effective. With the low incidence of intracranial disease in this group of patients, the impact of IT chemoprophylaxis is likely to be small and has to be weighed against morbidity associated with such therapy.

### ACKNOWLEDGEMENTS

The authors would like to thank Mrs CR Radstone and Dr PC Lorigan for their assistance in the conduct of this study.

#### REFERENCES

Althanassiou A, Newlands ES, Rustin GJS, et al (1983) Central nervous system metastases of choriocarcinoma: 23 years experience at Charing Cross Hospital. *Cancer* **52**: 1728–1735

Bagshawe KD and Harland S (1976) Immunodiagnosis and monitoring of gonadotrophin producing metastases in the central nervous system. *Cancer* **38**: 112–118

Brace KC (1968) The role of irradiation in the treatment of metastatic trophoblastic disease. *Radiology* **91**: 540–544

Du Beshter B, Berkowitz RS, Goldstein DP and Bernstein MR Management of low risk metastatic gestational trophoblastic tumour. *J Reprod Med* **36**: 36–39

Evans AC, Soper JT, Clarke-Pearson DL, Berchuck A, Rodriguez GC and Hammond CB (1995) Gestational trophoblastic disease metastatic to the central nervous system. *Gynecol Oncol* **59**(2): 226–230

Fisher PM and Hancock BW (1997) Gestational trophoblastic diseases and their treatment. *Cancer Treat Rev* **23**: 1–6

Newlands ES (1997) Investigation and treatment of patients with persistent Gestational Trophoblastic Disease and Gestational Trophoblastic Tumors in the UK. In *Gestational Trophoblastic Disease*, Hancock BW, Newlands ES and Berkowitz RS (eds), pp. 173–190. Chapman & Hall: London

Roberts JP and Lurain JR (1996) Treatment of low risk metastatic gestational trophoblastic tumour with single-agent chemotherapy. *Am J Obstet Gynecol* **174**: 1917–1924

Rustin GJS, Newlands ES, Begent RHJ, Dent J and Bagshawe KD (1989) Weekly alternating etoposide, methotrexate and actinomycin/vincristine and

© Cancer Research Campaign 1999

cyclophosphamide chemotherapy for the treatment of CNS metastases of choriocarcinoma. *J Clin Oncol* **7**: 900–903

Shapiro WR, Young DR and Mehta BM (1975) Distribution in cerebrospinal fluid after intravenous ventricular and lumbar injections. *NEJM* **293**: 161–166

Sheridan E, Hancock BW, Smith SC, Dorreen MS, Neal FE, Pennington GW and Millar DR (1993) Gestational trophoblastic disease: experience of the Sheffield (United Kingdom) supraregional screening and treatment service. *Int J Oncol* **3**: 149–155

Soper JT, Clarke-Pearson DL, Berchuck A, Rodriguez G and Hammond CB (1994). Five-day methotrexate for women with metastatic gestational trophoblastic disease. *Gynecol Oncol* **54**: 76–79

Weed JC and Hammond CB (1980) Cerebral metastatic choriocarcinoma: intensive therapy and prognosis. *Obstet Gynecol* **55**: 89–94

Yordan EL, Schlaerth J, Gaddis O and Morrow CP (1987) Radiation therapy in the management of gestational choriocarcinoma metastatic to the central nervous system. *Obstet Gynecol* **69**: 627–630

*© Cancer Research Campaign 1999*
